# Interface densification in a microphase-separated diblock copolymer resolved by small-angle X-ray scattering

**DOI:** 10.1107/S1600576725002638

**Published:** 2025-05-12

**Authors:** Yu-Hsuan Lin, Yu-Chung Chen, Aditya Sahare, Yi-Cheng Lai, Chun-Jen Su, Jing-Cherng Tsai, Katsuhiro Yamamoto, Hsin-Lung Chen

**Affiliations:** a Department of Chemical Engineering National United University Miaoli360302 Taiwan; b Department of Chemical Engineering National Tsing Hua University Hsinchu300044 Taiwan; c National Synchrotron Radiation Research Center Hsinchu300092 Taiwan; d Department of Chemical Engineering National Chung Cheng University Chiayi621301 Taiwan; e Department of Life Science and Applied Chemistry Nagoya Institute of Technology Nagoya Aichi466-8555 Japan; NSRRC, Taiwan

**Keywords:** block copolymers, interfaces, lower critical ordering transitions, electron density profiles, segmental densification

## Abstract

In block copolymers exhibiting lower critical ordering transition behavior, the segregation strength increases with temperature due to the significantly different thermal expansivities of the constituent blocks. Using synchrotron small-angle X-ray scattering, we reveal a phenomenon of segmental densification at the microdomain interface in this type of system, where the local electron density in a specific region of the interface surpasses that of the denser domain. As a result, the interfacial electron density profile deviates significantly from the hyperbolic tangent function expected for an incompressible melt. This unique densification effect can be attributed to the negative volume of mixing at the interface between the dissimilar segments and emerges when the electron densities of the constituent blocks are similar.

## Abstract 2

The electron density distribution along the lamellar normal in a block co­poly­mer, as determined by small-angle X-ray scattering, reveals segmental densification at the domain interface. This interfacial densification is attributed to the negative mixing volume in the segmental mixture of the constituent blocks, a distinct characteristic of block copolymers exhibiting lower critical ordering transition or hourglass phase behavior.

## 1. Introduction

Block copolymers constitute a fascinating class of soft material whose main feature lies in their capability of self-assembling into a broad spectrum of long-range-ordered nanostructures and their suitability for serving as a model system for examining the fundamental theories of soft matter (Bates & Fredrickson, 1990 ▸; Kim *et al.*, 2010 ▸; Leibler, 1980 ▸). One of the most notable instances of the latter is the verification of the Brazovskii universality class by demonstrating that the mean-field critical point of the block copolymer is replaced by a fluctuation-induced first-order transition that separates a periodically ordered phase from a disordered phase with spinodal-like composition fluctuations (Bates & Fredrickson, 1990 ▸; Bates *et al.*, 1990 ▸; Bates *et al.*, 1988 ▸; Fredrickson & Helfand, 1987 ▸).

The theories of AB diblock copolymers have been well developed for predicting the thermodynamic stabilities of various mesophases and the spinodal lines. A and B blocks in the diblock copolymers treated conventionally are energetically repulsive but entropically attractive, since microphase separation causes stretching and confinement of the block chains as well as the localization of the junction points at the interface, resulting in losses of conformational and translational entropy. As the weighting of the entropic contribution to free energy becomes greater at higher temperature, an order–disorder transition (ODT) occurs on heating and the copolymer is said to exhibit the ‘upper critical ordering transition’ (UCOT) behavior (Hamley & Hamley, 1998 ▸; Matsen & Bates, 1996 ▸; Ruzette & Leibler, 2005 ▸).

There is another class of diblock copolymer which shows the opposite temperature dependence of the segregation strength, namely, the net repulsion between A and B monomers becomes stronger at higher temperature. Here the ODT takes place on cooling and the copolymer is said to display the ‘lower critical ordering transition’ (LCOT) (Ahn *et al.*, 2013 ▸; Mulhearn & Register, 2017 ▸; Russell *et al.*, 1994 ▸; Ruzette *et al.*, 1998 ▸; Ryu *et al.*, 2002 ▸; Yeh *et al.*, 2011 ▸; Zhang *et al.*, 2020 ▸). The LCOT of diblock copolymers was first identified by Russell *et al.* (1994 ▸) and was attributed to the disparity in the thermal expansivities of the constituent blocks, which is of identical origin to that of the homopolymer blends showing a lower critical solution temperature (LCST) phase diagram (Russell *et al.*, 1994 ▸). In this case, A and B blocks could be energetically attractive but the constraint of the free volume of the more expansive component by the less expansive one results in an entropy loss when they are mixed with each other. As a result, a repulsive force with entropic origin develops to trigger the microphase separation at the elevated temperature. Predicting the LCOT phase behavior necessitates incorporating the compressibility of its constituent blocks into the free energy formulation (Hino & Prausnitz, 1998 ▸; Ruzette *et al.*, 2001 ▸; Yeung *et al.*, 1994 ▸). The binodal curves of both UCOT and LCOT can coexist within a single system and may intersect, leading to an ‘hourglass’ (HG) phase diagram (Hino & Prausnitz, 1998 ▸) in which the segregation strength first decreases and then increases with increasing temperature without transitioning through a disordered state.

It is known that the phase separation in an immiscible mixture generates an interface with a diffuse density profile, wherein the density decays monotonically as one moves from the higher-density phase toward the lower-density one. The diffuseness stems from the mixing gradient of the two constituents in the interfacial region for minimizing the interfacial free energy (Hashimoto *et al.*, 1986 ▸). Due to the restriction in the length scale of phase separation imposed by the covalent connectivity, the interface generated in the block copolymer is far more abundant than that developed in the immiscible blends of the corresponding homopolymers; consequently, the interface plays an important role in governing the properties of microphase-separated block copolymers.

Theoretical and experimental efforts have been made to resolve the interface structure of diblock copolymers (Anastasiadis *et al.*, 1990 ▸; Helfand, 1975*a* ▸; Helfand & Wasserman, 1977 ▸; Kawasaki *et al.*, 1988 ▸; Ohta & Kawasaki, 1986 ▸). The interfacial density profile represented by the number density of A segments was predicted to follow the hyperbolic tangent function under the incompressibility condition asserting that the sum of the local segmental densities of A and B blocks remains constant throughout the interface (Anastasiadis *et al.*, 1990 ▸; Helfand, 1975*a* ▸; Helfand & Wasserman, 1977 ▸; Kawasaki *et al.*, 1988 ▸; Ohta & Kawasaki, 1986 ▸). Under this condition, the interfacial free energy was considered to be composed of the following components: (1) the contact energy between dissimilar segments, (2) the loss of conformational entropy arising from the constraint that the A (B) block is not allowed to enter the B (A) microphase, and (3) the loss of conformational entropy of both blocks for attaining constant total segmental density (Helfand, 1975*b* ▸). The effect of the volume of mixing becomes significant in the systems showing LCOT or HG phase behavior; therefore, the entropic component originally associated with the melt incompressibility should be modified by including an additional entropic term accounting for the entropy loss of the more expansive constituent at the interface. It is hence of great interest to resolve whether the interfacial density profile is strongly perturbed from the hyperbolic tangent form in the microphase-separated diblock copolymers exhibiting LCOT or HG phase behavior.

In this study, we uncover an anomalous electron density profile of the interface which deviates from the hyperbolic tangent function in a lamella-forming diblock copolymer exhibiting a stronger segregation strength at higher temperature. We will show that the marked difference in thermal expansivity between the copolymer constituents led to a rapid diminishment of the primary scattering peak observed in the small-angle X-ray scattering (SAXS) profiles on cooling. This phenomenon primarily reflected the reduced electron density contrast between the two microphases and was not directly related to the onset of the ODT. By constructing the 1D electron density profile along the lamellar normal, we observed a densification phenomenon at the interface at sufficiently low temperatures, where the electron density in a specific region of the interface exceeded that of the microdomain core. This interface densification was attributed to the negative volume of mixing, where the specific volume of the mixture of the dissimilar segments at the interface was lower than that predicted by the rule of linear additivity.

## 2. Experimental

### 2.1. Materials and sample preparation

The diblock copolymer studied was a poly(ethyl­ene oxide)-*block*-poly(4-vinyl­pyridine) (PEO-*b*-P4VP) with number average molecular weights of the PEO and P4VP blocks of 5000 and 7200 g mol^−1^, respectively, and a polydispersity index of 1.28 (Polymer Source Inc.). The overall volume fraction of the PEO block was *ca* 0.39.

The as-received PEO-*b*-P4VP was dissolved in chloro­form to obtain a homogeneous solution by stirring at 45°C. The solution was then poured onto a petri dish, and most of the solvent was allowed to evaporate at room temperature for 48 h to form the as-cast film. Finally, the as-cast film was dried under vacuum at 40°C for 72 h to remove the residual solvent.

### 2.2. Small-angle X-ray scattering measurements

The temperature-dependent SAXS measurements were performed at beamline 23A of the National Synchrotron Radiation Research Center, Hsinchu, Taiwan. The energy of the X-ray source was 15 keV. The scattering signals were collected on a Pilatus-1MF detector of 981 × 1043 pixel resolution. The scattering intensity profile was output as the plot of the scattering intensity (*I*) versus the scattering vector magnitude *q* = (4π/λ)sin(θ/2) (where λ and θ are the X-ray wavelength and the scattering angle, respectively) after corrections for transmission and background.

For the temperature-dependent SAXS study, the sample with a thickness of 0.5 mm was first heated stepwise from 30 to 200°C to remove any prior thermal history. In this process, the sample was annealed above the *T*_g_ of P4VP (≃ 128°C), as measured by differential scanning calorimetry (DSC, see Fig. S1 in the supporting information) for approximately 90 min before the cooling process began. SAXS profiles were then collected at designated temperatures during stepwise cooling. At each temperature, the sample was equilibrated for 5 min, before 30 s of data acquisition.

### 2.3. PVT measurements

The specific volumes of PEO and P4VP were measured at different temperatures by a piston-type PVT instrument (GOTECH PVT-6000). An isobaric cooling procedure with the cooling rate 5°C min^−1^ was adopted to obtain the specific volume as a function of temperature in the cooling process. The testing procedure was standardized in ISO 17744:2004. The specific volume measurement was conducted at different pressures greater than the atmospheric pressure. The measured temperature ranged from 30 to 200°C. The PVT data of the samples at the atmospheric pressure were obtained by Tait model fitting. For an isothermal compressibility model (*i.e.* a volume–pressure relationship), the Tait equation is given by (Rodgers, 1993 ▸; Zoller & Fakhreddine, 1994 ▸)
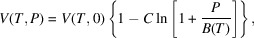
where *V*(*T*, 0) is the specific volume at zero gauge pressure, *C* is usually taken as a universal constant equal to 0.0894 and *B*(*T*) is the Tait parameter given by
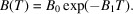


## 3. Results and discussion

### 3.1. Peculiar temperature-dependent SAXS pattern of the lamellar phase of PEO-*b*-P4VP

Our previous study demonstrated that poly(ethyl­ene oxide)-*block*-poly(2-vinyl pyridine) (PEO-*b*-P2VP) displayed the LCOT phase diagram (Yeh *et al.*, 2011 ▸). As a chemical analog of PEO-*b*-P2VP, PEO-*b*-P4VP was expected to show similar phase behavior. This was confirmed in a blend of PEO-*b*-P4VP and a P4VP homopolymer with an overall PEO volume fraction of 0.32, where an order–order transition from lamellar to cylindrical morphology was observed on cooling (see Fig. S2). This transition occurred in the opposite direction to what is observed in the UCOT systems, confirming that PEO-*b*-P4VP exhibited LCOT- or HG-type phase behavior, where the segregation strength decreased with decreasing temperature.

**Figure 1:**
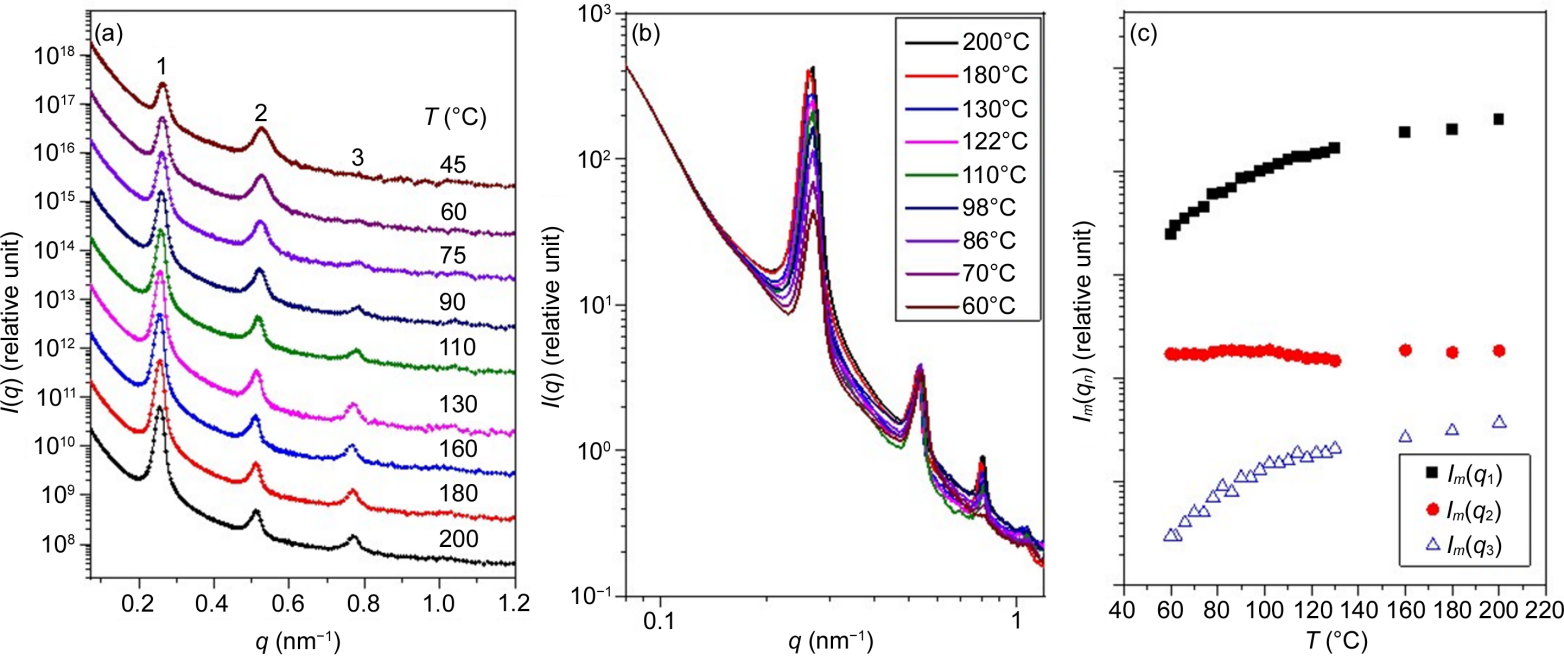
(*a*) Temperature-dependent SAXS profiles of PEO-*b*-P4VP collected in a cooling cycle. The scattering curves are shifted vertically for clarity. (*b*) Temperature-dependent SAXS profiles of PEO-*b*-P4VP without vertical shift. (*c*) Plot of the integrated peak intensities as a function of temperature, where *I_m_*(*q*_1_), *I_m_*(*q*_2_) and *I_m_*(*q*_3_) denote the intensity of the first-, second- and third-order peaks, respectively.

Fig. 1 ▸(*a*) presents the temperature-dependent SAXS profiles of PEO-*b*-P4VP collected during the cooling process. No crystallization of PEO was observed over the displayed temperature range. At 200°C, the SAXS profile exhibited three peaks with a position ratio of 1:2:3, confirming the formation of a lamellar structure with an interlamellar distance (*d*) of 24.74 nm. As the temperature decreased, the interlamellar distance progressively decreased (Fig. S3), aligning with an increase in the effective segregation strength of PEO-*b*-P4VP, a characteristic feature of LCOT phase behavior.

The intensity of the primary peak also diminished with decreasing temperature, which might initially suggest a weakening of segregation strength. However, as will be shown later, this reduction in peak intensity primarily resulted from a decrease in electron density contrast between the PEO and P4VP microdomains, arising from differences in their thermal expansivities. Consequently, caution is required when using primary peak intensity variation to determine the ODT temperature (*T*_ODT_). Furthermore, while an ODT was expected during cooling, it was effectively inaccessible in PEO-*b*-P4VP. The relatively high *T*_g_ of the P4VP block led to the vitrification of the P4VP domain before the system reached *T*_ODT_, thereby preventing the transition to the disordered state.

The most notable feature of the SAXS profiles in Fig. 1 ▸(*a*) is the different temperature dependences of the individual peak intensities. The first- and the third-order peaks diminished progressively with decreasing temperature, whereas the intensity of the second-order peak remained virtually unperturbed. This phenomenon was manifested clearly in the SAXS profiles without vertical shift [see Fig. 1 ▸(*b*)] and the plot of the integrated peak intensities as a function of temperature shown in Fig. 1 ▸(*c*).

Note that the temperature-dependent SAXS results presented in Fig. 1 ▸ are reliable, despite the relatively short equilibration time of approximately 5.5 min at each temperature during the cooling process. This reliability was confirmed by a similar cooling experiment conducted using an in-house SAXS instrument, where a much longer equilibration time of 40 min yielded nearly identical results (see Fig. S4).

### 3.2. The origin of the peculiar temperature dependence of scattering peak intensities

The intensities of the diffraction peaks of a lamellar structure are known to depend on the volume fractions of the individual layers. For a two-phase lamellar structure with varying layer thickness, the intensity of the *n*th-order peak is related to the electron density contrast Δρ_e_ and layer volume fraction *f*_1_ via *I*_m_(*q_n_*) ≃ Δρ_e_^2^ sin^2^(*n*π*f*_1_)/*n*^4^ (Roe, 2000 ▸). Fig. S5 presents the temperature-dependent relative peak intensities of PEO-*b*-P4VP, calculated using the PEO volume fraction and the electron density contrast derived from the specific volumes of PEO and P4VP homopolymers, as measured by dilatometry (see Fig. 4). All three peaks showed a similar trend, with their intensities decreasing as the temperature decreased. The predicted temperature variation of the second-order peak intensity did not align with the experimental observations, which showed that the intensity of this peak remained largely unchanged. This indicates that the observed temperature variations in peak intensities in Fig. 1 ▸ cannot be attributed solely to the change in volume fraction within the lamellar structure.

**Figure 2:**
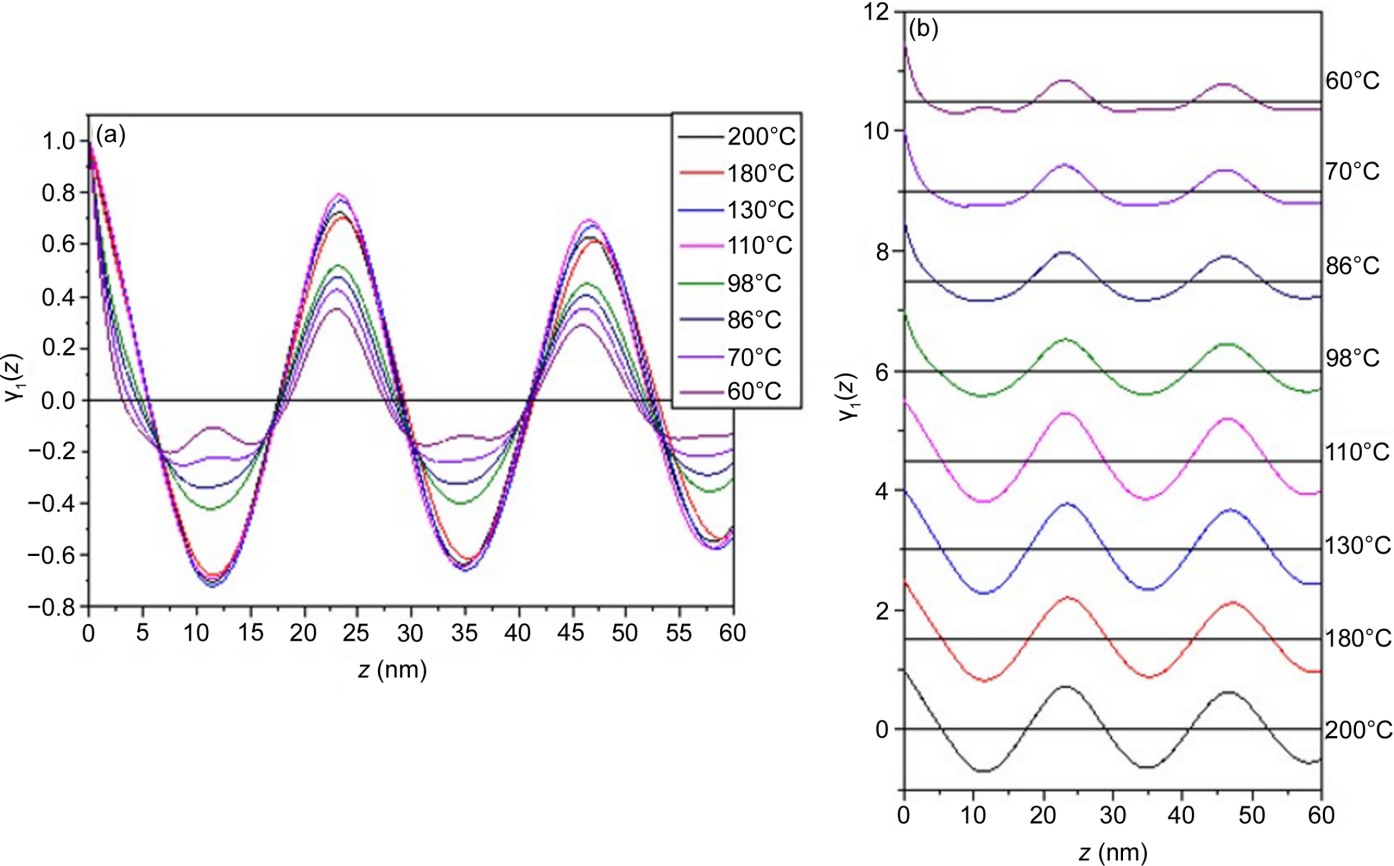
(*a*) Temperature-dependent 1D correlation function of PEO-*b*-P4VP. (*b*) 1D correlation functions shifted vertically for clarity. At *T* ≥ 110°C, γ_1_(*z*) exhibited regular oscillation prescribed by the typical two-phase lamellar structure. The shape of γ_1_(*z*) showed obvious change at temperatures lower than 110°C, where an additional small peak situating at *z* ≃ *d*/2 emerged.

To investigate the origin of the unusual temperature dependence of peak intensities, we calculated the normalized 1D electron density correlation function along the lamellar normal (*z* direction) using the Fourier cosine transform of the scattering intensity, *viz.* (Roe, 2000 ▸)
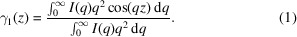
Fig. 2 ▸ displays the correlation functions obtained for different temperatures. At elevated temperatures (*e.g.* 200°C), γ_1_(*z*) exhibited regular oscillations consistent with the two-phase lamellar structure, where the periodic variation in electron density was confined by the finite grain size (Roe, 2000 ▸; Ruland, 1977 ▸). The location of the first maximum of γ_1_(*z*) corresponds to the interlamellar distance. As the temperature decreased, the amplitude of oscillation of γ_1_(*z*) reduced and its profile began to show significant differences particularly when the temperature dropped below 110°C, where the self-correlation triangle at *z* < 10 nm became curved. An additional small peak, located at approximately *z* ≃ *d*/2, gradually emerged during cooling and was distinctly visible at 60°C.

**Figure 3:**
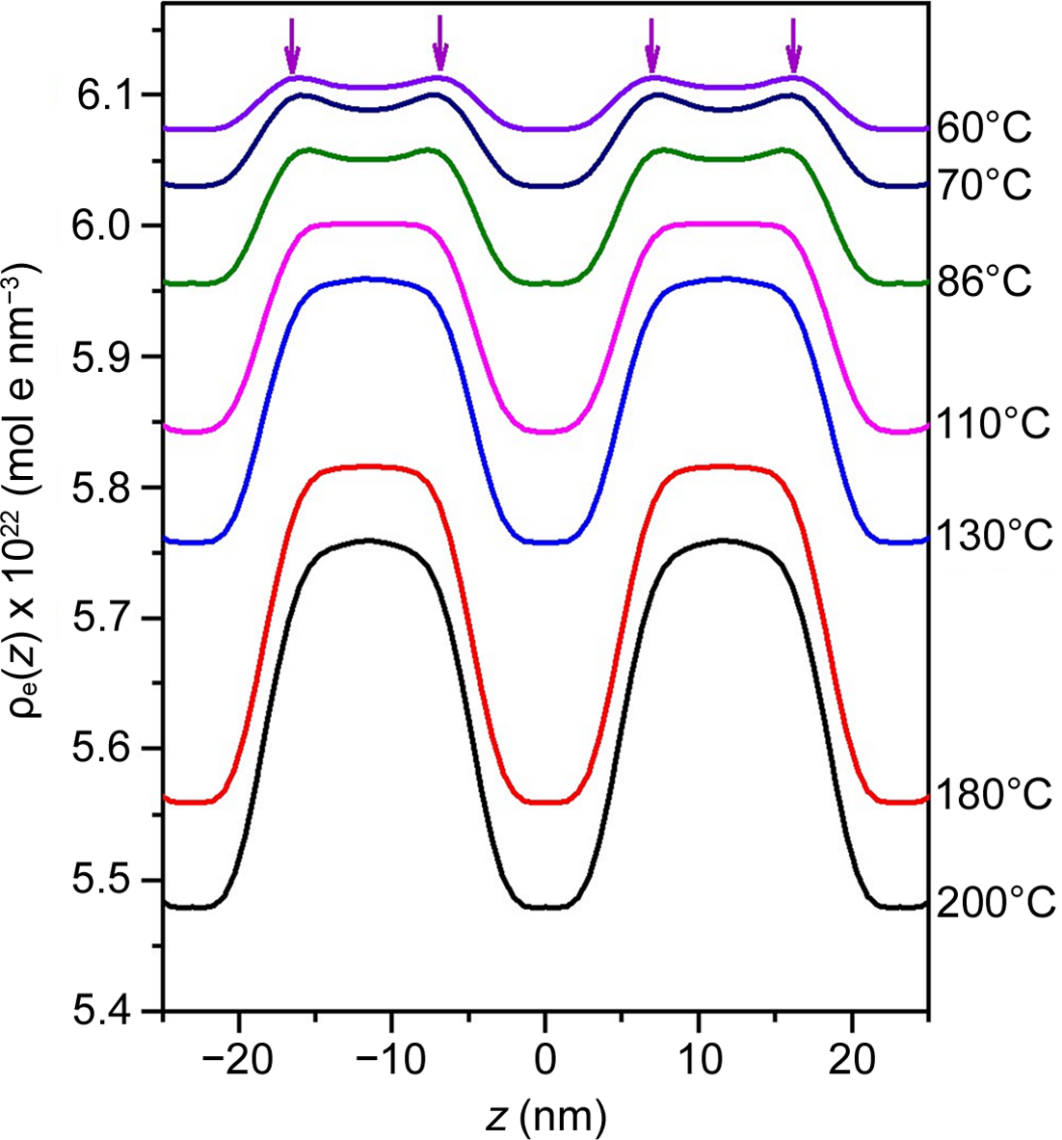
Electron density profiles along the lamellar normal at different temperatures. The electron density is presented on an absolute scale, converted from the electron density data of PEO and P4VP in Fig. 4(*b*). The electron density profile above 110°C could be described by the two-phase model with a diffuse interface, the region with the lower and the higher electron density corresponding to the PEO and P4VP domains, respectively. The shape of the electron density profile at *T* ≤ 86°C showed a three-phase characteristic.

Given that γ_1_(*z*) represents the convolution of the 1D electron density profile, ρ_e_(*z*), the changes in the shape of γ_1_(*z*) indicate that the electron density profile of the lamellar structure deviated significantly from the traditional two-phase model below 110°C. To investigate this further, we reconstructed the relative electron density profile along the lamellar normal by superimposing the Fourier modes associated with the observed scattering peaks, *viz.* (Chang *et al.*, 2011 ▸; Wachtel *et al.*, 1998 ▸; Wu *et al.*, 2004 ▸)
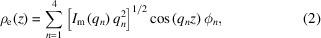
where *q_n_* and *I*_m_(*q_n_*) are the position and intensity of the *n*th-order peak, respectively; ϕ*_n_* is the corresponding phase whose value is either +1 or −1 for the centrosymmetric system; and [*I*_m_(*q_n_*)*q_n_*^2^]^1/2^ represents the amplitude of the *n*th Fourier mode. Four observed scattering peaks were used to calculate the electron density profiles, where the phase assignment of (−1, −1, +1, +1) was found to yield the most reasonable ρ_e_(*z*) for the lamellar structure, as shown in Fig. 3 ▸. The electron density profiles above 110°C were well described by a two-phase model with a diffuse interface, where the regions of lower and higher electron density correspond to the PEO and P4VP domains, respectively. The electron density at the interface decreases monotonically as one moves from the P4VP domain to the PEO domain along the *z* direction.

**Figure 4:**
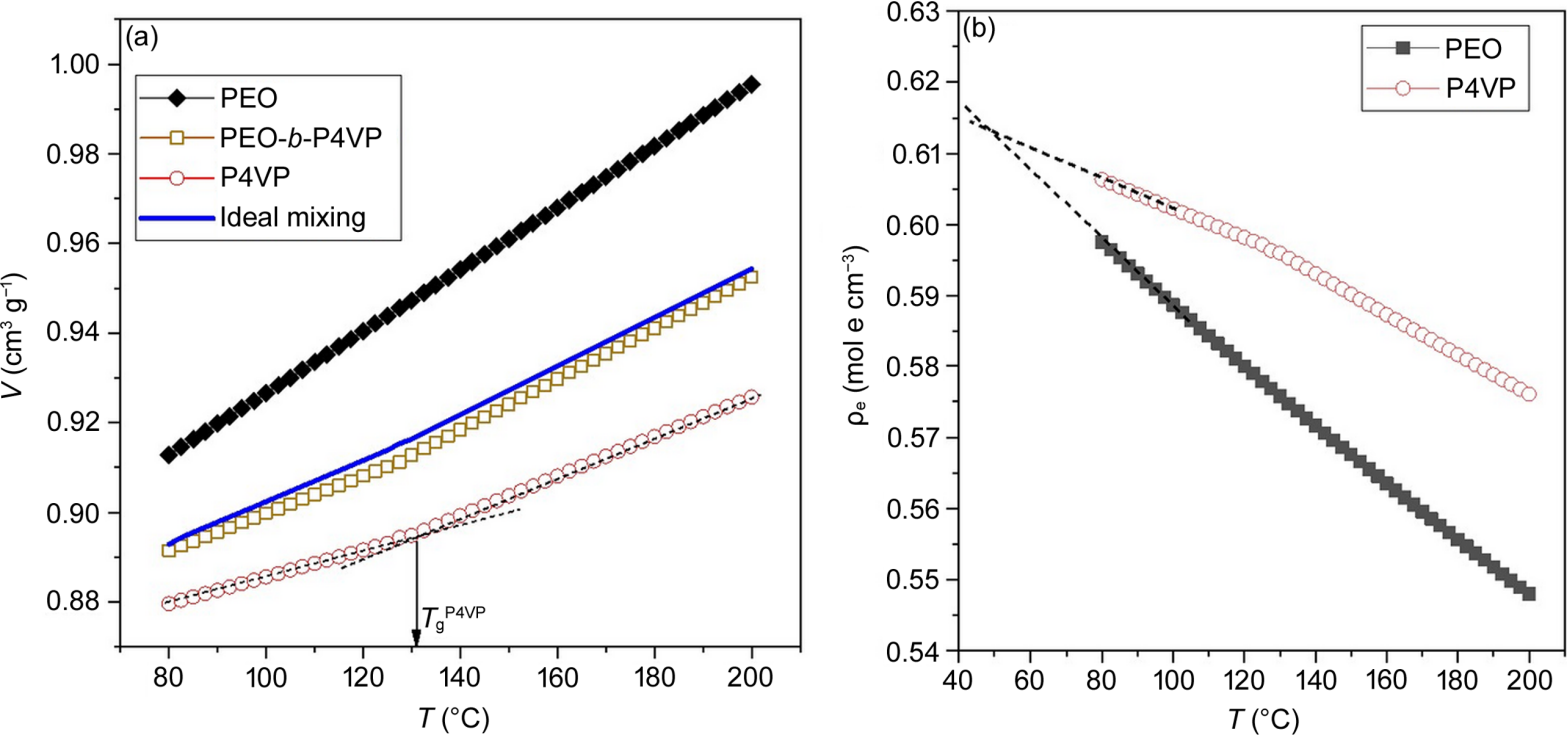
(*a*) Temperature dependence of the specific volumes of the P4VP homopolymer, PEO homopolymer and PEO-*b*-P4VP at 1 bar obtained by the Tail model. The number-average molecular weights and polydispersity indices of the PEO and P4VP homopolymers are 5000 (g mol^−1^)/1.12 and 7000 (g mol^−1^)/1.04, respectively, which are very similar to those of the PEO and P4VP blocks in the copolymer. The blue line corresponds to the calculated specific volume of the miscible mixture of PEO and P4VP assuming ideal mixing. (*b*) Electron densities of PEO and P4VP as a function of temperature calculated from the specific volume data in (*a*). The electron density is given by ρ_e_ = ρ(*n*_me_/*M*_m_), where ρ, *n*_me_ and *M*_m_ are the mass density, the number of moles of electrons per mole of repeating unit and the molecular weight of the repeating unit, respectively. For PEO, *n*_me,EO_ = 24 mol e mol^−1^ and *M*_m,EO_ = 44 g mol^−1^. For P4VP, *n*_me,4VP_ = 56 mol e mol^−1^ and *M*_m,4VP_ = 105 g mol^−1^.

The amplitude of ρ_e_(*z*) progressively decreased as the temperature was lowered, indicating a reduction in electron density contrast during cooling. This decrease in contrast was attributed to the significant difference in thermal expansion coefficients between PEO and P4VP, which resulted in a smaller density difference between the two components at lower temperatures. Fig. 4 ▸(*a*) presents the specific volumes of these two homopolymers as a function of temperature measured by dilatometry. The disparity in thermal expansion coefficient was evidenced by the different slopes, with the specific volume of PEO increasing more rapidly than that of P4VP as the temperature increased. The thermal expansion coefficient of PEO was determined to be 7.55 × 10^−4^ K^−1^. For P4VP, a change in slope was observed around 131°C, corresponding to its glass transition temperature *T*_g_. This *T*_g_ for P4VP aligned with the value measured by DSC. The thermal expansion coefficients of P4VP in the glassy and rubbery states were 3.38 × 10^−4^ and 4.93 × 10^−4^ K^−1^, respectively.

The measured specific volumes were used to calculate the electron densities of the two polymers as a function of temperature, as shown in Fig. 4 ▸(*b*). The electron density contrast, given by the difference in ρ_e_ between P4VP and PEO, decreased with decreasing temperature. The match point was identified at approximately 48°C; however, the SAXS profile at 45°C still exhibited clear peaks. This discrepancy can be attributed to differences in cooling rates between the dilatometry and SAXS experiments. Specifically, in the dilatometry experiment, the sample was cooled from 100 to 60°C within 5 min, whereas the SAXS measurement spanned approximately 60 min over the same temperature range. As a result, the non-equilibrium glassy P4VP domain had more time to relax during the SAXS experiment, leading to a higher mass density and, consequently, a greater electron density contrast compared with the dilatometry measurement.

Additionally, a complete matching of electron density between the two microphases is inherently challenging due to the presence of the interface, which introduces a subtle but persistent density variation. This interfacial effect further contributes to the incomplete cancellation of the lamellar peaks in SAXS, even at temperatures where the electron densities of PEO and P4VP microdomains appear to match according to dilatometry data.

The decrease in Δρ_e_ with lowering temperature, as demonstrated in Fig. 4 ▸(*b*), suggested that the weakening of the primary scattering peak during cooling was primarily caused by the reduction of the contrast instead of the onset of an ODT. This hypothesis was confirmed by comparing the temperature dependence of the primary peak intensity with that of the square of the electron density contrast, Δρ_e_^2^, as shown in Fig. S6. The nearly parallel variations of the peak intensity and contrast factor confirmed our hypothesis.

As Δρ_e_ continued to decrease with cooling, the shape of the electron density profile also changed, as shown in Fig. 3 ▸(*b*). Below 110°C, two humps (highlighted by arrows) appeared beside a valley in the higher-density domain, giving the overall electron density profile a three-phase characteristic. These humps originated from the increased contribution of the second-order peak intensity to the Fourier mode [*cf.* equation (2)] relative to the first-order peak at lower temperatures. Consequently, the peculiar temperature dependence of the peak intensities observed in Fig. 1 ▸ can be attributed to the transformation of the electron density profile from a two-phase to a more three-phase-like structure during cooling.

**Figure 5:**
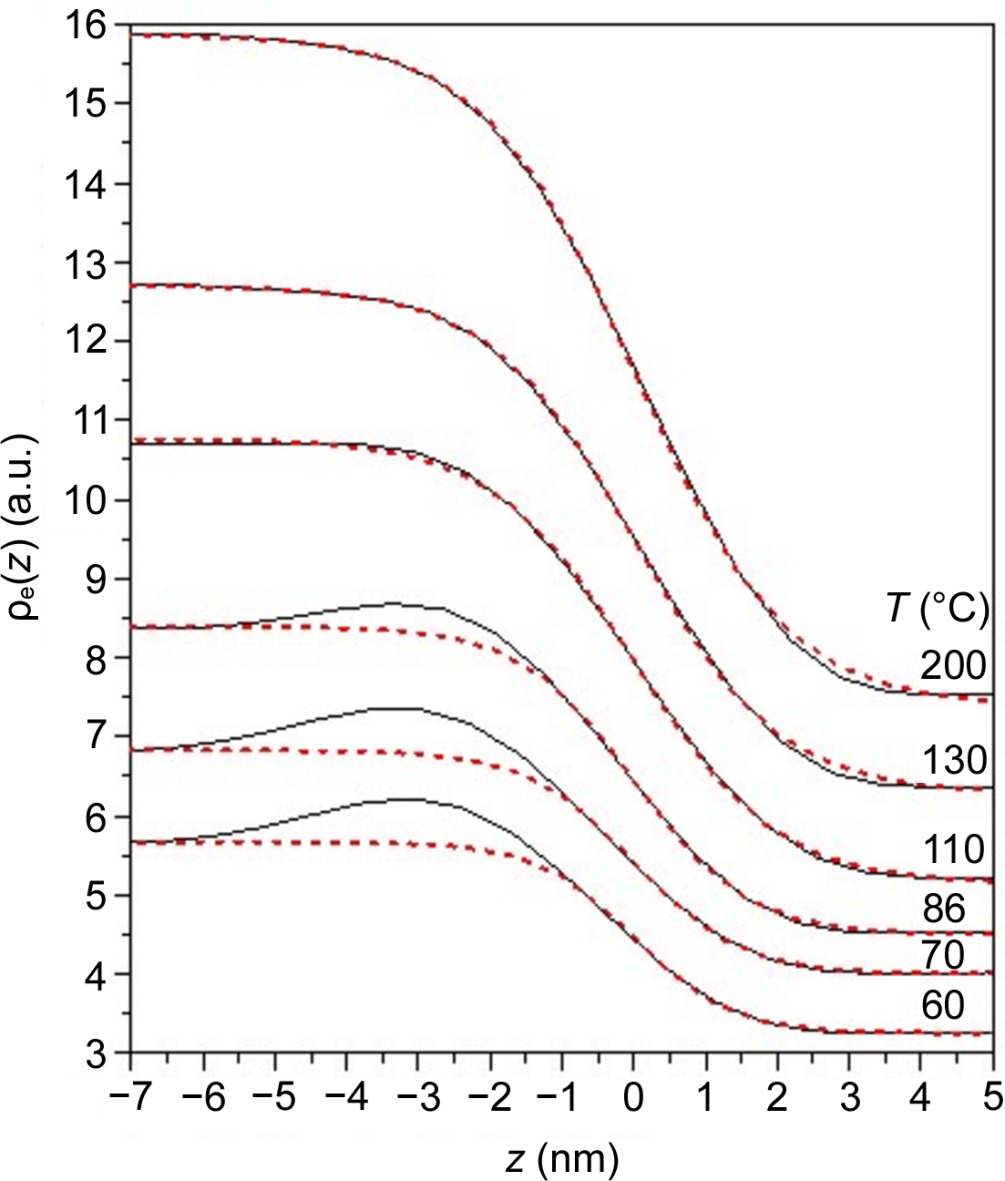
Interfacial electron density profiles on relative scales at the temperatures indicated in the figure. The red dashed curves are the fits by equation (4) using the hyperbolic tangent in equation (5). The electron density curves have been rescaled for better visualization of the fitting results.

Fig. 5 ▸ shows the ρ_e_(*z*) profile across the interface enlarged from Fig. 3 ▸. The electron density distribution at the interface is directly related to the segmental density distributions of the constituent blocks via
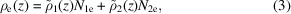
where 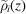 is the local segmental density of component *i* (*i.e.* the number of *i* segments per unit volume) and *N*_*i*e_ is the number of electrons per segment. Under the incompressibility condition, 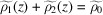, equation (3) is rewritten as
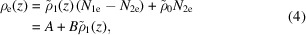
where *A* and *B* are constants independent of *z*. Equation (4) shows that the shape of the electron density profile is governed by the segmental density profile of component 1 in an incompressible melt, which is described by the hyperbolic tangent function, *viz.* (Anastasiadis *et al.*, 1990 ▸; Helfand & Sapse, 1975 ▸)
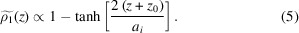
Here *z*_0_ is the location of the center of the interface and *a_i_* is the thickness of the interface. The dashed curves in Fig. 5 ▸ correspond to the fits based on equations (4) and (5), where each profile was shifted by its respective *z*_0_, as determined from the fit along the *z* axis, aligning the interface center at *z* = 0. It is evident that the hyperbolic tangent function provided a satisfactory fit to the interfacial density profile at *T* ≥ 110°C

At *T* ≤ 86°C, the electron density profiles showed a clear deviation from the hyperbolic tangent function represented by the dashed curves. In this case, the hump observed in the overall electron density profile indicates a region within the interface where the electron density exceeded that of the P4VP microdomain core. This suggests that a segmental densification occurred in the interfacial region at sufficiently low temperatures, giving the overall electron density profile a three-phase-like characteristic.

Table S1 of the supporting information lists the values of the interface thickness *a_i_* associated with the dashed curves obtained from the fitting. The interface thickness appeared to decrease with lowering temperature. This behavior was attributed to the tendency of the enhancement of the electron density at the interface due to the occurrence of densification as the temperature decreased.

### 3.3. Origin of the interface densification evidenced by the electron density profile

Given that PEO has a larger thermal expansion coefficient, its free volume should increase more rapidly than that of P4VP on heating, amplifying the difference in free volume between the two components. If PEO and P4VP mix at the segmental level, the component with the higher free volume (*i.e.* PEO) would be condensed by the other, resulting in a negative volume of mixing and a loss of entropy. This negative excess entropic contribution to the free energy becomes more significant at higher temperatures, thereby enhancing the segregation strength.

Although P4VP and PEO blocks were segregated, their segmental mixing inevitably occurred at the interface, leading to a negative volume of mixing in this region. As shown in Fig. 4 ▸, the measured specific volume of PEO-*b*-P4VP at a given temperature was slightly smaller than the value predicted by the rule of linear additivity (represented by the solid line) for ideal mixing according to the specific volumes of the pure components. This deviation suggests the presence of a negative volume of mixing at the interface, and such an effect could explain the densification phenomenon observed in the electron density profile of the interface.

As a first-order approximation, we assume that in a unit weight of the mixture the reduction in free volume per PEO–P4VP segmental contact is *g*_f_. Therefore, the volume of mixing is −*g*_f_*w*_1_*w*_2_, where the product of the weight fractions *w*_1_*w*_2_ represents the probability of 1–2 contact under a mean-field approximation. The specific volume of the mixture can thus be expressed as
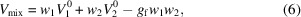
where 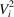 is the specific volume of pure constituent *i*. The local electron density ρ_e_(*z*) is related to the local mass density of the mixture ρ_mix_(*z*) and the local weight fractions of the constituents *w_i_*(*z*) via
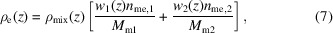
where *M*_m,*i*_ and *n*_me,*i*_ are the molecular weight and the moles of electrons per mole of the repeating unit of component *i*, respectively. For PEO-*b*-P4VP, *n*_me,EO_ = 24 mol e mol^−1^, *M*_m,EO_ = 44 g mol^−1^, *n*_me,4VP_ = 56 mol e mol^−1^ and *M*_m,4VP_ = 105 g mol^−1^.

To resolve the conditions under which the negative volume of mixing could lead to the segmental densification reflected in the electron density profile, we calculated the electron density profile using equation (7), with an assumed weight fraction profile for the lamellar structure and an arbitrary value of *g*_f_ = 0.01, incorporating the specific volume values of PEO and P4VP at 200 and 60°C.

**Figure 6:**
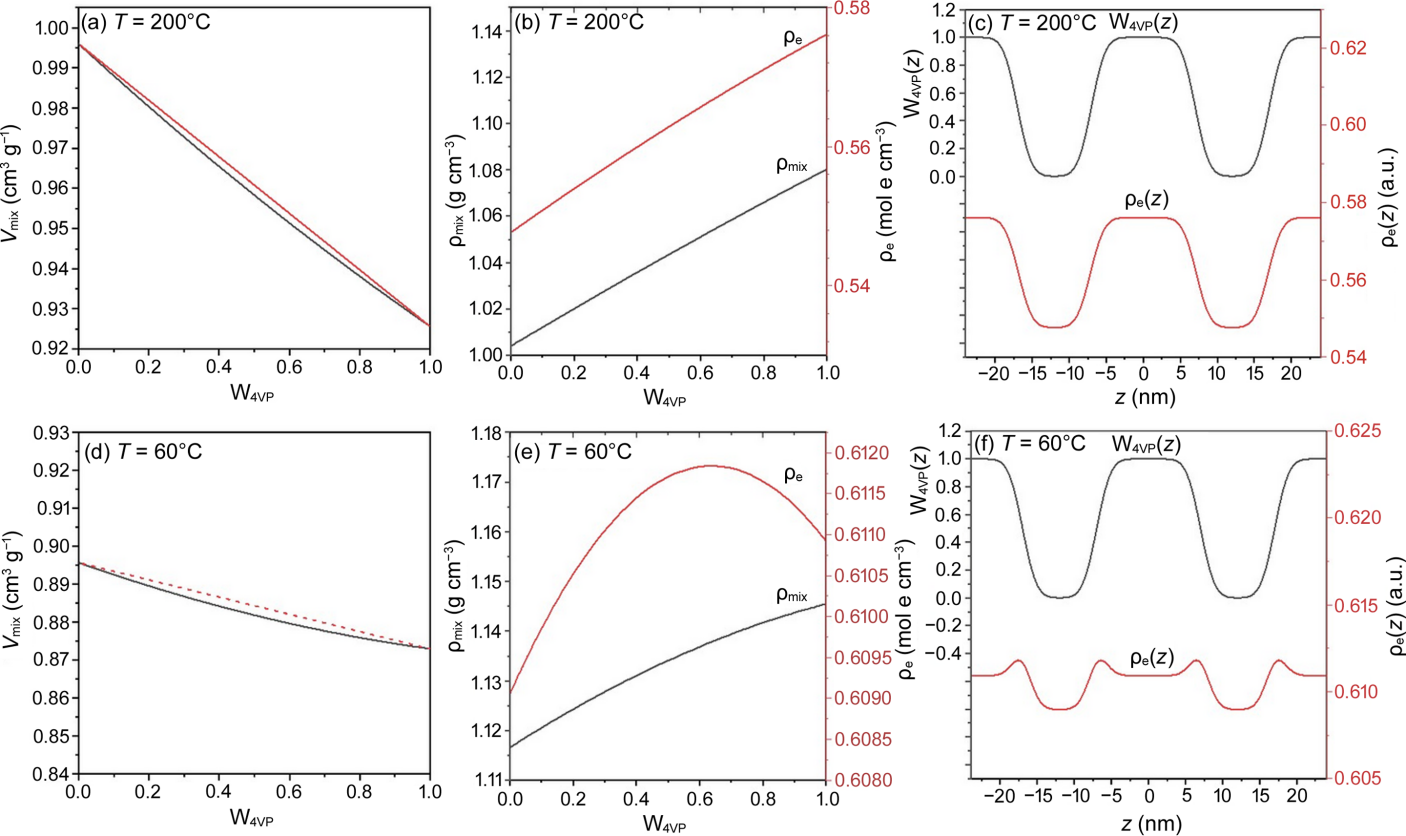
(*a*) Specific volume of the segmental mixture of PEO and P4VP at 200°C calculated by equation (6), assuming *g*_f_ = 0.01. (*b*) Corresponding composition variation of the mass density and electron density of the PEO/P4VP mixture at 200°C. (*c*) Corresponding electron density profile calculated from an assumed weight fraction profile *w*_4VP_(*z*) shown in the plot. The electron density profile still shows the two-phase characteristic. (*d*) Specific volume of the segmental mixture of PEO and P4VP at 60°C calculated by equation (6), assuming *g*_f_ = 0.01. (*e*) Corresponding composition variation of the mass density and electron density of the PEO/P4VP mixture at 60°C. (*d*) Corresponding electron density profile calculated from an assumed weight fraction profile *w*_4VP_(*z*). The electron density profile now exhibits a three-phase characteristic with two humps located in the vicinity of the P4VP microdomain.

According to Fig. 4 ▸, the specific volumes of PEO and P4VP at 200°C were 0.996 and 0.926 cm^3^ g^−1^, respectively. The *V*_mix_ values of the PEO/P4VP mixture calculated with *g*_f_ = 0.01, as displayed in Fig. 6 ▸(*a*), showed a negative excess volume of mixing, where *V*_mix_ was smaller than that predicted by the rule of linear additivity. Both the mass density and the electron density of the mixture were found to increase monotonically with the increase of *w*_4VP_, as shown in Fig. 6 ▸(*b*).

Knowing the composition-dependent mass density of the mixture, the electron density profile, ρ_e_(*z*), of the lamellar structure can be calculated using equation (7), based on the spatial variation of the P4VP weight fraction, *w*_4VP_(*z*). *w*_4VP_(*z*) was assumed to follow a two-phase model with a diffuse interface, as illustrated in Fig. 6 ▸(*c*). The resulting ρ_e_(*z*) was found to exhibit a two-phase characteristic [see Fig. 6 ▸(*c*)]. The phenomenon of interfacial densification, where the electron density at the interface surpassed that of the microdomain cores, was not observed.

At 60°C, the specific volumes of PEO and P4VP were 0.896 and 0.873 cm^3^ g^−1^, respectively. As shown in Figs. 6 ▸(*d*) and 6 ▸(*e*), while the calculated values of *V*_mix_ and ρ_mix_ (with *g*_f_ = 0.01) decreased and increased monotonically with increasing P4VP weight fraction, respectively, the electron density displayed a maximum value near *w*_4VP_ = 0.64. This composition variation of ρ_e_ resulted in a densification effect in the ρ_e_(*z*) profile, calculated using the same *w*_4VP_(*z*) profile as for 200°C, as shown in Fig. 6 ▸(*f*). The ρ_e_(*z*) profile then exhibited a three-phase characteristic resembling the experimental profile at 60°C (see Fig. 3 ▸).

The above analysis demonstrated that the negative volume of mixing, which yielded a maximum ρ_e_ at the intermediate compositions, resulted in an enhancement of the electron density at the interface. However, a similar negative volume of mixing was also present at 200°C, but the ρ_e_ of the mixture increased monotonically with P4VP composition and thus did not produce the same densification effect. Below, we aim to demonstrate that the similarity in electron density (or mass density) between the two constituent blocks is a key factor driving the emergence of the interfacial densification in the electron density profile, in addition to the negative volume of mixing. In this context, the absence of the densification effect at 200°C can be attributed to the relatively large difference in electron density between PEO and P4VP at elevated temperatures.

Equation (7) can be expressed in terms of the electron densities of the two pure constituents by knowing that
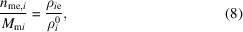
where ρ_*i*e_ and ρ_*i*_^0^ are the electron density and mass density of component *i*, respectively. Substituting into equation (7) yields
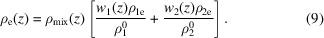
The interfacial densification results in the electron density at the interface exceeding that of the pure components; moreover, we let ρ_1e_ > ρ_2e_, and thus ρ_e_(*z*) >> ρ_1e_ > ρ_2e_. Then we have
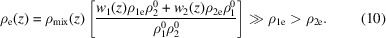
Since ρ_mix_(*z*) = *V*_mix_(*z*)^−1^ and ρ*_i_*^0^ = *V_i_*^0–1^, it can be shown from equation (10) that
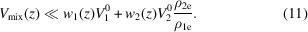
Substituting the expression of *V*_mix_(*z*) from equation (6) into equation (11), we obtain the condition necessary for observing significant interface densification in the electron density profile as
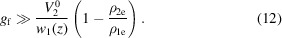
The criterion outlined by equation (12) can be easily met with a large *g*_f_ under a fixed ρ_2e_/ρ_1e_ or by allowing ρ_2e_/ρ_1e_ to approach 1 under a fixed *g*_f_. The first condition indicates a significant negative volume of mixing, while the second condition reflects similar electron densities between the two constituents. Fig. S7 illustrates both scenarios: the effect of the *g*_f_ value on the ρ_e_(*z*) profile with a fixed electron density ratio of ρ_2e_/ρ_1e_ = 0.973, and the effect of ρ_2e_/ρ_1e_ with a fixed *g*_f_ = 0.01. It is clear that interface densification in the electron density profile became more pronounced when *g*_f_ increased and ρ_2e_/ρ_1e_ approached 1.0. At 200°C, the electron densities of PEO and P4VP were significantly different, with ρ_2e_/ρ_1e_ = 0.9506, leading to the absence of the densification effect despite the presence of a negative volume of mixing. In contrast, at 60°C, the electron densities of the two components were sufficiently similar (ρ_2e_/ρ_1e_ = 0.973), allowing the densification effect to occur due to the attainment of maximum electron density at the intermediate composition within the interface.

Note that our analysis using the specific volume data at 200 and 60°C was not intended to provide any quantitative characterization of *g*_f_ (the volume of mixing) or to fully resolve the thermodynamics underlying the temperature-dependent interface structure in the PEO-*b*-P4VP system. Instead, the aim of the analysis was to clarify the origin of the electron density enhancement at the interface observed experimentally and to identify the conditions necessary for this phenomenon. The choice of these two temperatures was driven by the significant difference in the observed electron density profiles, which exhibited a two- or three-phase characteristic. Further application of our analysis to accurately elucidate the thermodynamic phase behavior and the equilibrium interface structure was constrained by the non-equilibrium nature of the lamellar morphology below *T*_g_^P4VP^. At these temperatures (*e.g.* 60°C), the P4VP microdomain resided in a non-equilibrium glassy state, and the interface was likely kinetically arrested and hence deviated from equilibrium.

Nevertheless, this study has highlighted the enhancement in electron density at the interface of the lamellar structure of PEO-*b*-P4VP, which is a distinctive characteristic of the diblock copolymer displaying LCOT or HG phase behavior. This densification effect becomes pronounced when the volume of mixing is significantly negative and the electron densities of the two components are similar, the conditions that are typically met at lower temperatures. Both conditions are closely linked to the substantial difference in the thermal expansivities of the constituent blocks.

## 4. Conclusions

In this study, we investigated the interface structure of the lamella-forming PEO-*b*-P4VP. The temperature-dependent SAXS results demonstrated a decrease in the intensity of the primary scattering peak during cooling, while the intensity of the second-order peak remained largely unchanged. The significant reduction in the primary peak intensity at lower temperatures was attributed to a decrease in the electron density contrast between the two microphases, rather than the occurrence of an ODT. This behavior was primarily due to the substantial difference in the thermal expansivity between the constituent blocks.

The construction of the electron density profile along the lamellar normal revealed a densification phenomenon at the microdomain interface at sufficiently low temperatures, where the electron density at the interface exceeded that of the P4VP domain. Consequently, the electron density profile exhibited a three-phase characteristic. The observed transformation of the electron density profile from a two-phase to a three-phase characteristic accounted for the distinctive temperature dependence of the SAXS peak intensities during the cooling process.

The interface densification was linked to the negative volume of mixing in the segmental mixture of PEO and P4VP, a unique feature of the diblock copolymer exhibiting LCOT or HG phase behavior. Thermodynamically, gradient mixing of the PEO and P4VP segments occurred at the interface, resulting in a hyperbolic tangent distribution of the weight fraction of the two segments in the interface. Under the conditions of (1) negative volume of mixing and (2) similar electron densities of the two constituents, this hyperbolic tangent profile translated into an electron density profile in which the electron density at the interface exceeded those of the PEO and P4VP microdomains.

## Supplementary Material

Supporting figures. DOI: 10.1107/S1600576725002638/ju5085sup1.pdf

## Data Availability

The supporting information includes a DSC thermogram for measuring the thermal transitions of PEO-*b*-P4VP; the order–order transition from the lamellar structure to the hexagonally packed cylinder morphology in a blend of PEO-*b*-P4VP with P4VP homopolymer in the cooling process to demonstrate the LCOT behavior of PEO-*b*-P4VP; the temperature dependence of the interlamellar distance of PEO-*b*-P4VP; temperature-dependent SAXS profiles of PEO-*b*-P4VP measured with longer equilibration times using an in-house SAXS instrument; the temperature dependence of the SAXS peak intensities calculated from the electron density contrast and the layer volume fraction of the lamellar structure; comparison between the temperature variation of the first-order peak intensity and the electron density contrast factor; the values of the interface thickness obtained by fitting the observed electron density profile of the interface using equations (4) and [Disp-formula fd7](5); the effects of the free volume condensation factor, *g*_f_, and the ratio of the electron densities of the constituent blocks ρ_2e_/ρ_1e_ on the electron density profile of the interface.
