# Supplementary material for: Interface densification in a microphase-separated diblock copolymer resolved by small-angle X-ray scattering
Source: J Appl Crystallogr. 2025 May 12;58(Pt 3):869–78. doi: 10.1107/S1600576725002638 (PMC12135978; doi:10.1107/S1600576725002638)
Supplement: Supplementary file 1 [file j-58-00869-sup1.pdf]

JOURNAL OF APPLIED CRYSTALLOGRAPHY

Volume 58 (2025)

Supporting information for article:

Interface densification in microphase-separated diblock copolymer resolved by small-angle X-ray scattering

Yu-Hsuan Lin, Yu-Chung Chen, Aditya Sahare, Yi-Cheng Lai, Chun-Jen Su, Jing-Cherng Tsai, Katsuhiro Yamamoto and Hsin-Lung Chen

1. Differential scanning calorimetry (DSC) thermogram for measuring the thermal transitions of the PEO-*b*-P4VP.

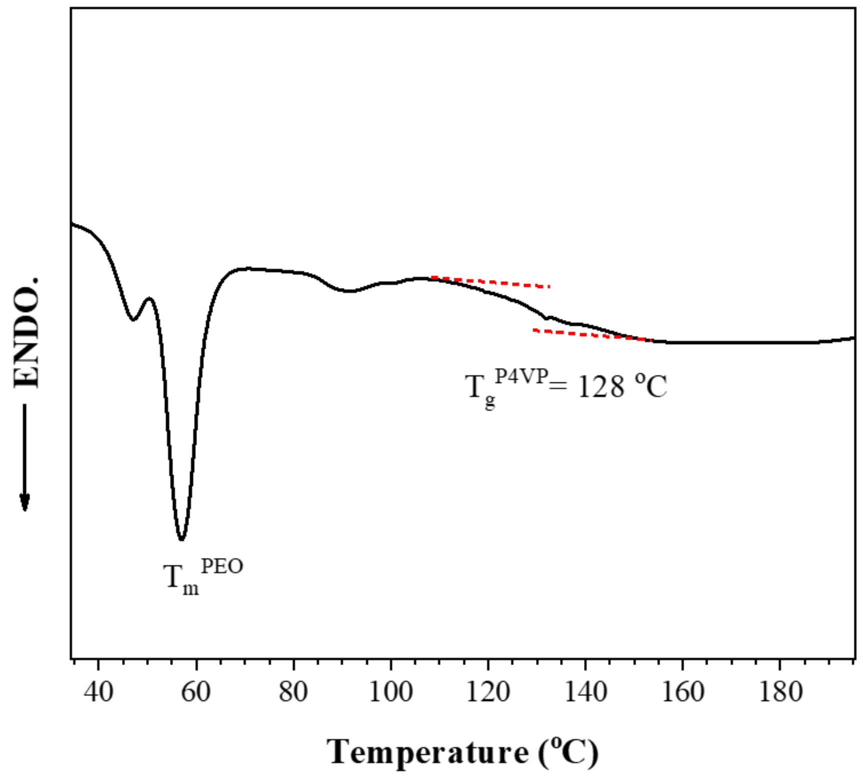

Fig. S1. The DSC thermogram of the PEO-*b*-P4VP studied. The measurement was conducted using a Netzsch DSC 200 F3 differential scanning calorimeter. The sample was pre-heated to 200 °C at a rate of 10 °C/min, held at this temperature for five minutes, and then cooled to 10 °C at the same rate. Data were collected during a subsequent heating scan at 10 °C/min. The thermogram reveals a melting endotherm of the crystals formed by PEO block at approximately 57 °C and the glass transition temperature (T_g) of P4VP block at 128 °C.

2. Order-order transition from lamellar (*LAM*) structure to hexagonally-packed cylinder (*HEX*) morphology in a blend of PEO-*b*-P4VP with P4VP homopolymer in the cooling process to demonstrate the lower critical ordering transition (LCOT) behavior of PEO-*b*-P4VP.

The poly(ethylene oxide)-*block*-poly(4-vinylpyridine) (PEO-*b*-P4VP) with the molecular weight of M_{bEO} = 4000 g/mol and M_{b4VP} = 6000 g/mol (PDI=1.16) was blended with a P4VP homopolymer with molecular weight of M_{h4VP} = 1000 g/mol (PDI=1.20) to yield the blend with the overall P4VP weight fraction of 0.68. Fig. S2 shows the temperauture-dependent SAXS profiles of the blend. The SAXS profile at 180 ºC displayed two peaks with the position ratio of 1:2, signifying the formation of *LAM* morphology with D = 18.6 nm in the blend. As can be seen from enlarged SAXS profiles in Fig. S2(a), the lamellar structure persisted from 180 to 120 ºC in the cooling process. As the temperature was lowered to 110 ºC, a tiny scattering peak with the position of $(3)^{1/2}q_1$ was seemingly identified, as shown in Fig. S2(b). This peak grew progressively on further cooling, as demonstrated in Fig. S2(c). At 45 and 60 ºC, three diffraction peaks with the position ratio of 1 : $(3)^{1/2}$: 2 were clearly discernible, indicating the formation of *HEX* morphology formed by the blend. On basis of the temperature-dependent SAXS result, an OOT from *LAM* to *HEX* in the cooling process was revealed. This direction of phase transition was opposite to that displayed by the conventional UCOT diblocks; it hence offers a solid evidence for the decrease of segregation strength with decreasing temperature for PEO-*b*-P4VP system.

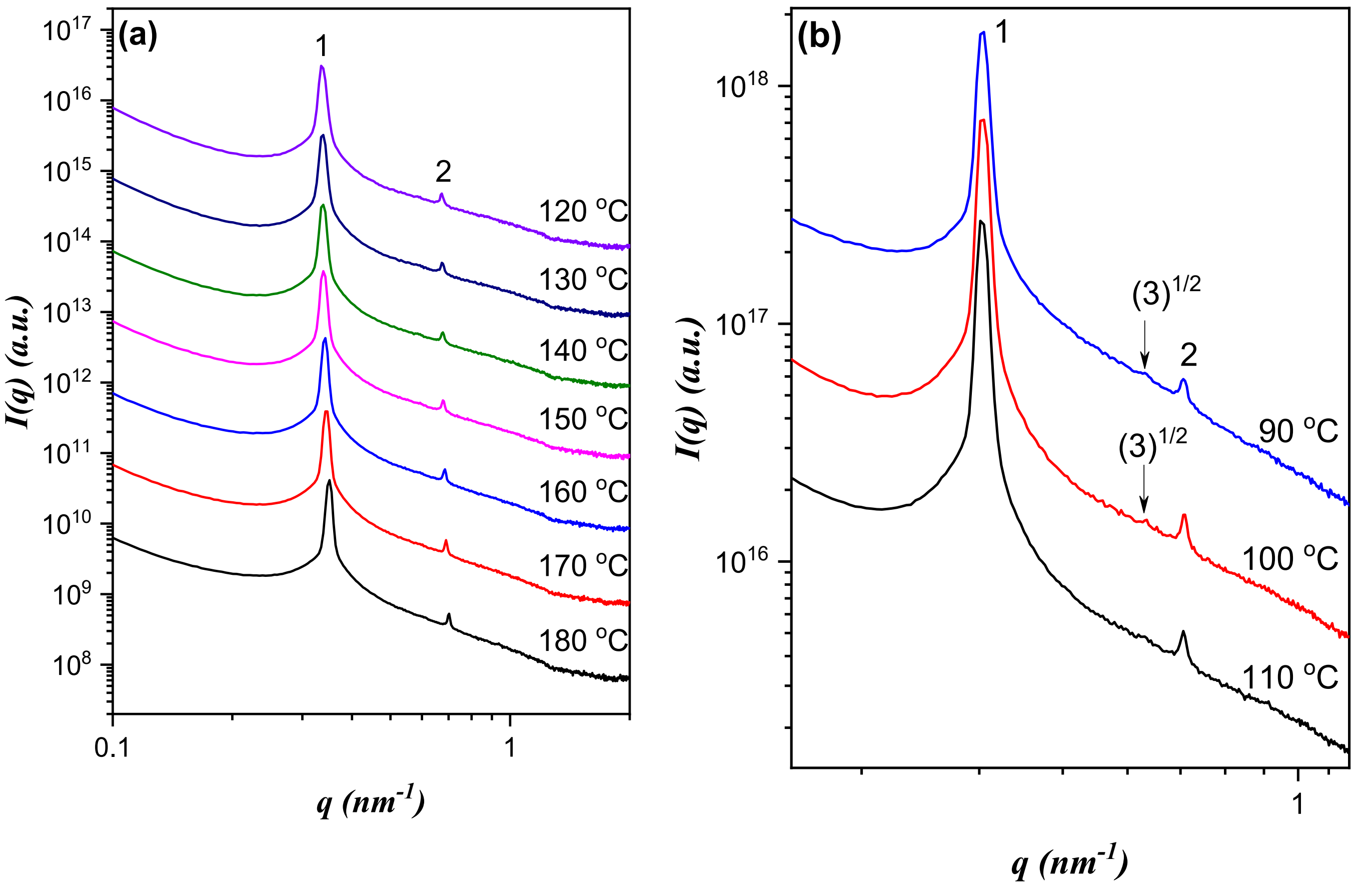

Fig. S2. Temperature-dependent SAXS profiles of the PEO-*b*-P4VP/P4VP blend with the overall weight fraction of P4VP w_{4VP} = 0.68 collected in a cooling cycle **(a)** from 180 to 120 ºC, **(b)** from 110 to 90 ºC and **(c)** from 75 to 45 ºC.

3. Temperature dependence of the interlamellar distance of PEO-*b*-P4VP

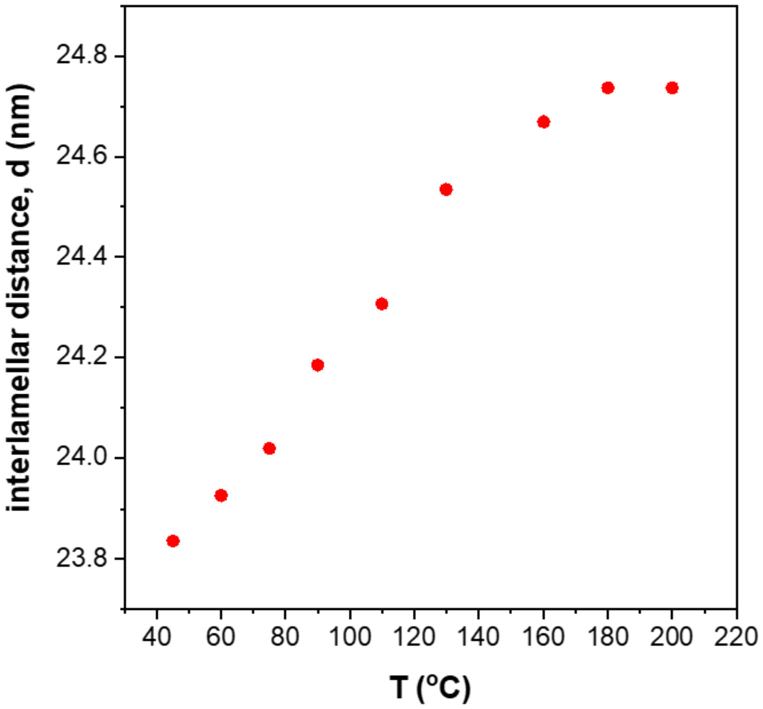

Fig. S3. Temperature dependence of the interlamellar distance of PEO-*b*-P4VP calculated from the position of the first-order peak (q_1) via $d = 2\pi/q_1$. The interlamellar distance increased with increasing temperature, suggesting an enhancement in the effective segregation strength of the block copolymer, a characteristic signature of LCOT phase behavior.

4. Temperature-dependent SAXS profiles of PEO-*b*-P4VP measured with longer equilibration time using an in-house SAXS instrument.

To verify the reliability and reproducibility of the SAXS results in Fig. 1, which were collected with a relatively short equilibration time (ca. 5.5 min), we conducted the temperature-dependent SAXS measurements using an in-house SAXS instrument. The experiments were conducted with a Bruker N8 Horizon SAXS instrument in a cooling cycle. In this setup, the sample was annealed at each temperature for 30 minutes, followed by 10 minutes of data acquisition, giving a total annealing time of 40 minutes.

The SAXS instrument was furnished with a IμS micro-focus X-ray generator operated at 50 kV×1000 μA. The wavelength of the X-ray source λ was 0.154 nm. Data were collected using a VANTEC-500 area detector with 2048×2048 pixel resolution located at 663.6 mm from the sample producing a q range of 0.1 ~ 3.5 nm^{-1}, where $q = 4\pi\sin(\theta/2)/\lambda$ with θ being the scattering angle. The collected scattering patterns were radially averaged to obtain one-dimensional scattering intensity profiles. All the scattering profiles were corrected for the scatterings from air and cell.

Fig. S4 presents the resulting SAXS profiles and the corresponding temperature dependence of the peak intensities. It can be seen that the in-house SAXS results closely align with those reported in Fig. 1, which were obtained using synchrotron radiation SAXS with shorter annealing times. Specifically, the intensities of the first- and third-order peaks decrease progressively as the temperature decreases, while the second-order peak intensity remains largely unchanged. This consistency confirms the reliability and reproducibility of the experimental data presented in Fig. 1.

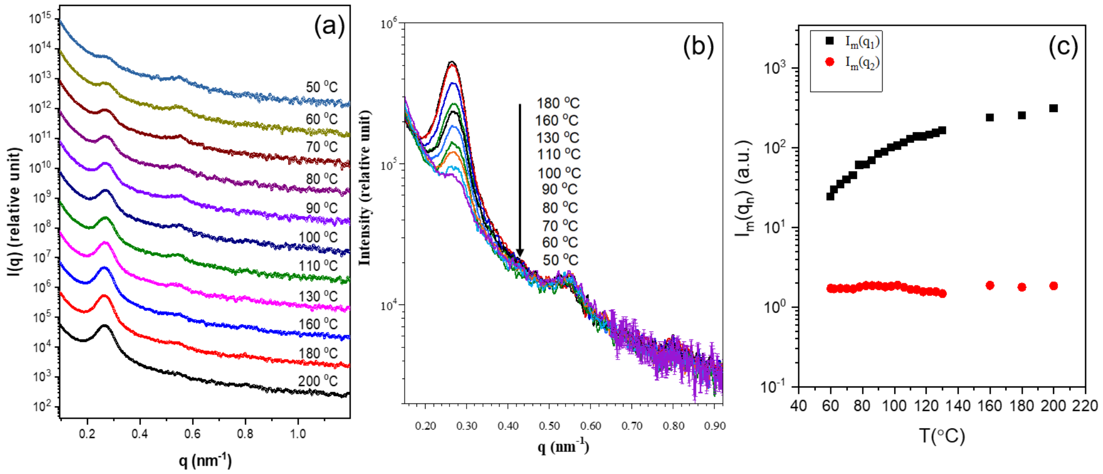

Fig. S4. (a) Temperature-dependent SAXS profiles of the PEO-*b*-P4VP collected during a cooling process using an in-house SAXS instrument. The sample was annealed at each temperature for 30 minutes, followed by 10 minutes of data acquisition, giving a total annealing time of 40 minutes. The scattering curves are shifted vertically for the clarity of presentation. **(b)** Temperature-dependent SAXS profiles without vertical shift. **(c)** The plot of the integrated peak intensities as a function of temperature, where $I_m(q_1)$, $I_m(q_2)$ and $I_m(q_3)$ denote the intensity of the first-, second-, and third-order peak, respectively.

5. The temperature dependence of the SAXS peak intensities calculated from the electron density contrast and the layer volume fraction of the lamellar structure.

We calculated the intensities of the scattering peaks according to the layer volume fractions and the electron density contrasts at different temperatures and compare the temperature dependence of the calculated intensities with that observed in Fig. 1.

For a two-phase lamellar structure with varying layer thickness, the intensity of the nth-order peak is related to the electron density contrast $\Delta\rho_e$ and layer volume fraction f_1 via

$$I_m(q_n) \sim \Delta\rho_e^2 sin^2(n\pi f_1)/n^4.$$

Fig. S5 presents the temperature-dependent relative peak intensities of PEO-*b*-P4VP, calculated using the PEO volume fraction and the electron density contrast derived from the specific volumes of PEO and P4VP homopolymers, as measured by dilatometry (Fig. 4). It is evident that all three peaks showed a similar trend, with their intensities decreasing as the temperature decreased. The predicted temperature variation of the second-order peak intensity does not align with experimental observations, which show that the intensity of this peak remains largely unchanged. This indicates that the observed temperature variations in peak intensities in Fig. 1 cannot be attributed solely to the change in volume fraction within the lamellar structure.

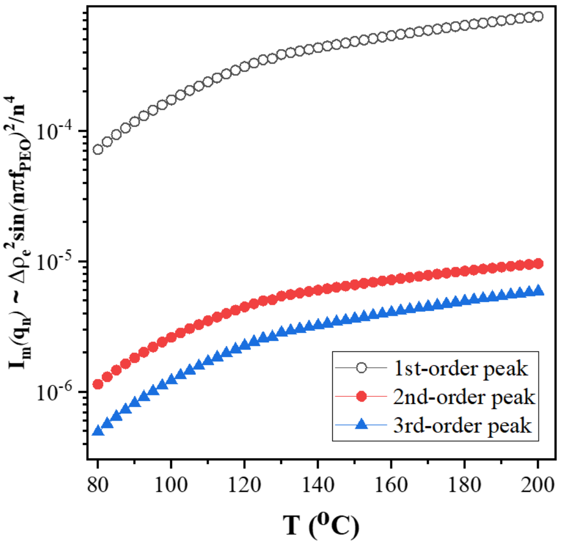

Fig. S5 The temperature-dependent relative peak intensities of PEO-*b*-P4VP, calculated using the PEO volume fraction and the electron density contrast derived from the specific volumes of PEO and P4VP homopolymers

Comparison between the temperature variation of the first-order peak intensity and electron density contrast factor

To verify the that reduction of the intensity of the first-order peak with decreasing temperature was due mainly to the reduction of electron density contrast, we compare the observed peak intensity with the square of the electron density contrast, $\Delta\rho_e^2$, as shown in Fig. S6. It can be seen that the variation of the primary peak intensity with temperature is nearly parallel to that of the contrast factor, confirming our hypothesis.

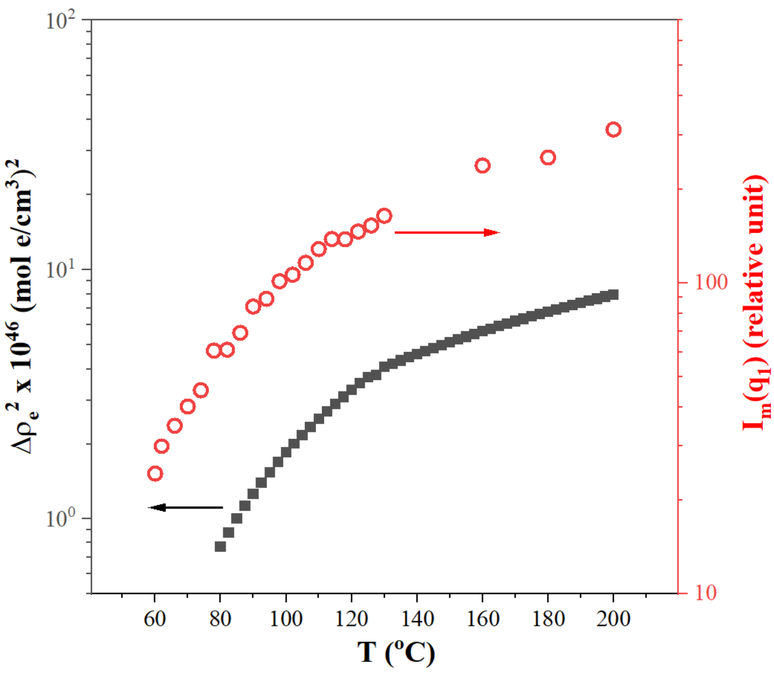

Fig. S6. Comparison of the temperature variation of the observed intensity of the primary peak and that of the contrast factor$\Delta\rho_e^2$.

6. The values of the interface thickness obtained by fitting the observed electron density profile of the interface using Eq. (4) and (5).

Table S1 The values of the interface thickness associated with the dashed curves obtained from the fitting using Eq. (4) and (5).

**Temperature (ºC)**	**interface thickness, a_i (nm)**
200	3.98 ± 0.10
130	3.85 ± 0.08
110	3.76 ± 0.08
86	3.07 ± 0.07
70	2.85 ± 0.07
60	2.67 ± 0.08

7. The effects of the free volume condensation factor, g_f, and the ratio of the electron densities of the constituent blocks r_{2e}/r_{1e} on the electron density profile of the interface.

Fig. S7 illustrates the effect of g_f on the electron density profile of the interface with a fixed electron density ratio of $\rho_{2e}/\rho_{1e} = 0.973$, and the effect of ρ_{2e}/ρ_{1e} with a fixed $g_f = 0.01$. It is clear that interface densification in the electron density profile becomes more pronounced when g_f increases and ρ_{2e}/ρ_{1e} approaches 1.0.

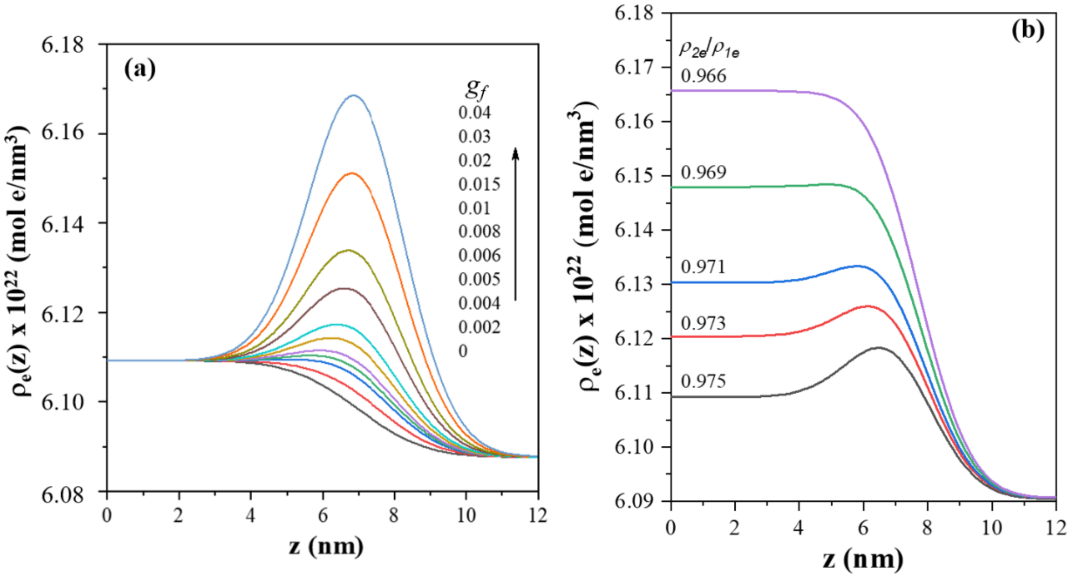

Fig. S7 The effects of the values of g_f and ρ_{2e}/ρ_{1e} on the electron density profile of the interface.
